# *Cis*‐unsaturated sphingolipids support growth of sphingoid base‐deficient yeast but impair plasma membrane integrity

**DOI:** 10.1002/2211-5463.70100

**Published:** 2025-08-05

**Authors:** Takashi Higuchi, Saki Sugihara, Yohei Ishibashi, Kono Yushi, Hazuki Yamauchi, Motohiro Tani

**Affiliations:** ^1^ Faculty of Applied Biological Sciences Gifu University Japan; ^2^ Department of Chemistry, Faculty of Sciences Kyushu University Fukuoka Japan; ^3^ The United Graduate School of Agricultural Science Gifu University Japan; ^4^ Graduate School of Bioresource and Bioenvironmental Sciences Kyushu University Fukuoka Japan

**Keywords:** complex sphingolipid, *Saccharomyces cerevisiae*, sphingoid long‐chain base, sphingolipid, ceramide

## Abstract

Sphingoid long‐chain bases (LCBs) form the backbone of sphingolipids, and their structures vary among eukaryotes. For example, in budding yeast, phytosphingosine is the major LCB, while animals primarily use sphingosine. Animals and plants also produce structurally diverse LCBs, including species with additional *cis* or *trans* double bonds, which are absent in yeast. Here, we show that yeast can grow even when its endogenous LCBs are replaced with plant‐type unsaturated forms, such as (8*Z*)‐4‐hydroxy‐8‐sphingenine or (4*E*,8*E*)‐sphinga‐4,8‐dienine. These cells synthesized ceramides and complex sphingolipids, indicating efficient incorporation of the exogenous LCBs into sphingolipid metabolism. However, cells harboring these unsaturated LCBs exhibited abnormalities in plasma membrane permeability, lipid order, and distribution of some plasma membrane‐localized proteins. In contrast, these cells reinforce their cell walls, presumably to compensate for the impaired plasma membrane integrity. Notably, to our knowledge, this is the first report of eukaryotic cells whose sphingolipids are composed almost exclusively of LCBs with a *cis* double bond, providing a unique model platform to investigate how LCB structural features influence membrane function.

## Graphical abstract

Sphingoid base structures, the sphingolipid backbones, vary among species. We established yeast cells in which the native sphingoid base was replaced with plant‐type bases containing *cis* or *trans* double bonds. This is, to our knowledge, the first eukaryotic model mostly composed of sphingolipids containing *cis*‐unsaturated sphingoid base, providing a unique platform to study how sphingoid base structure influences membrane function.

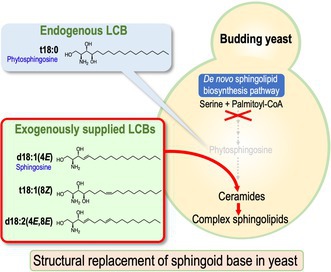

**Abbreviations**
AbA: aureobasidin A
Cer: ceramide
DHS: dihydrosphingosine
IPC: inositol phosphorylceramide
LCB: long‐chain base
M(IP)_2_C: mannosyldiinositol phosphorylceramide
MIPC: mannosylinositol phosphorylceramide
PHS: phytosphingosine
SPH: sphingosine

Sphingolipids are a class of lipids that have a sphingoid long‐chain base (LCB) as their fundamental backbone and are essential for the growth of eukaryotic organisms. Ceramides (Cers), which are *N*‐acylated derivatives of LCBs, and complex sphingolipids, which have hydrophilic head groups attached to Cers, play crucial roles in maintaining the localization and activity of membrane proteins by participating in the formation of lipid microdomains along with sterol molecules [1]. Although sphingolipids are conserved as membrane lipids in all eukaryotic cells, their structures vary among different organisms. In the budding yeast *Saccharomyces cerevisiae*, the LCB moiety of sphingolipids consists only of phytosphingosine (PHS) and dihydrosphingosine (DHS), with PHS being the predominant form [2] (Fig. 1A). In contrast, animals and plants have sphingosine (SPH), which is not present in *S. cerevisiae*, in addition to PHS and DHS [3, 4, 5]. In animals, SPH is the primary LCB structure [3]. In addition, animals and plants also have LCB structures with additional *trans* or *cis* double bonds, which are absent in *S. cerevisiae*. For example, in animals, (4*E*,14*Z*)‐sphinga‐4,14‐dienine (d18:2(4*E*,14*Z*)), which has a *cis* double bond at the C14 position of SPH, is present and is particularly abundant in the kidney [6]. In plants, structures with a *trans* or *cis* double bond at the C8 position of their LCBs have been identified (e.g., (8*Z*)‐4‐hydroxy‐8‐sphingenine (t18:1(8*Z*)), (8*E*)‐4‐hydroxy‐8‐sphingenine (t18:1(8*E*)), (4*E*,8*E*)‐sphinga‐4,8‐dienine (d18:2(4*E*8*E*)), and (4*E*,8*Z*)‐sphinga‐4,8‐dienine (d18:2(4*E*8*Z*))) [4, 5]. These differences in LCB structures among species are thought to have been acquired through evolution to enable adaptation to the environment and unique biological functions in each organism. In plants, for example, desaturation at the C8 position of LCBs has been shown to affect membrane fluidity and contribute to aluminum tolerance, representing a case in which LCB structural variation influences membrane function [7, 8]. Moreover, the presence of double bonds in LCBs is thought to significantly influence the ability of sphingolipids to participate in lipid microdomain formation. Specifically, while some sphingolipids in plants and animals contain *cis* double bonds, most eukaryotic sphingolipids lack such unsaturation, which is considered a key structural feature for the formation of lipid microdomains [1, 6]. Therefore, in mammalian cells, sphingolipids containing d18:2(4*E*,14*Z*) are excluded from such domains, which may suggest a physiological role for LCBs containing a *cis* double bond in the regulation of lipid microdomains [6]. However, the functional implications of sphingolipids containing *cis* or *trans* double bonds at positions like C8 or C14 are still not fully understood. Moreover, the biological significance of species‐specific structural differences in LCBs has yet to be fully elucidated.

**Fig. 1:**
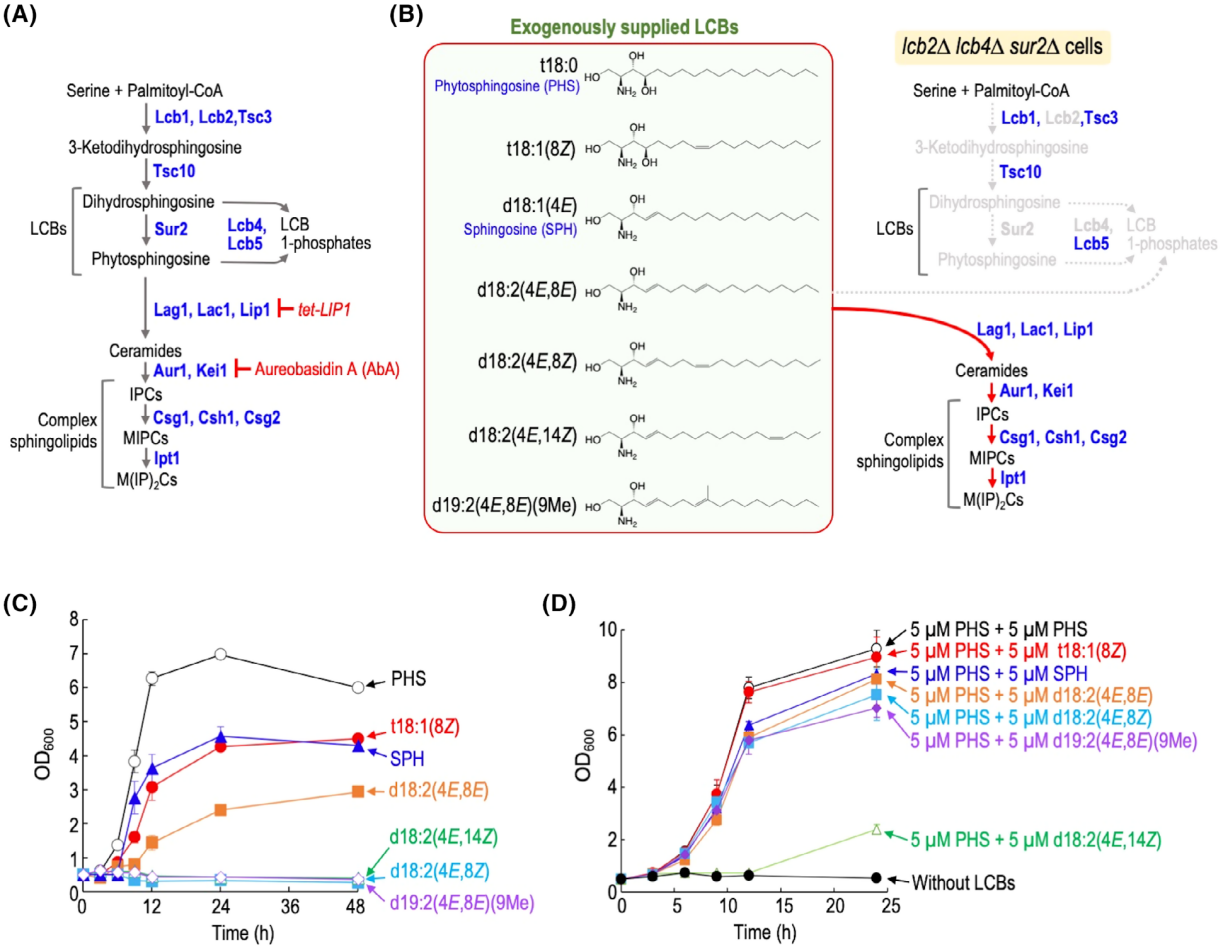
Structures of long‐chain base (LCB) replacements. (A) *De novo* biosynthesis pathway of complex sphingolipids in budding yeast. The proteins involved in each metabolic step are shown. Aureobasidin A (AbA) inhibits inositol phosphorylceramide (IPC) synthase Aur1 that catalyzes transfer of phosphoinositol from PI to Cers. For inhibition of Cer biosynthesis, expression of *LIP1* was repressed by the tetracycline‐regulatable promoter (*tet‐LIP1*). (B) Structures of LCB replacements. *LCB2‐*deleted cells cannot grow due to the inability of LCB biosynthesis; however, exogenously added LCBs, depending on their structure, can complement the growth of the mutant cells. Deletion of *LCB4* encoding major LCB kinase enables growth of SPH‐supplied *LCB2*‐deleted cells because of prevention of conversion of SPH to SPH 1‐phosphate. (C) Time course of cell growth of LCB‐supplied *lcb2∆ lcb4∆ sur2∆* cells. Cells were cultured at 30 °C in the presence of 5 μm each LCB, and aliquots of the cultures were taken to measure cell density (OD_600_) at the indicated times. Data represent means ± SD for one experiment (triplicate) representative of three independent experiments. (D) Effect of various LCBs on the growth of *lcb2∆ lcb4∆ sur2∆* cells in the presence of phytosphingosine (PHS). *lcb2∆ lcb4∆ sur2∆* cells were cultured at 30 °C with or without 5 μm PHS and 5 μm each LCB, and aliquots of the cultures were taken to measure cell density (OD_600_) at the indicated times. Data represent means ± SD for one experiment (triplicate) representative of three independent experiments.

In previous studies, it was found that *S. cerevisiae* can grow even when the LCB structure is almost entirely replaced by SPH (d18:1) [9, 10] (Fig. 1B). Briefly, deletion of serine palmitoyltransferase, which catalyzes the first step of sphingolipid biosynthesis, causes a lethal phenotype; however, growth can be complemented by supplementing with exogenous PHS or DHS [11]. In contrast, the addition of SPH fails to complement the cell growth because SPH is converted to SPH 1‐phosphate, which causes cytotoxicity [11, 12]. To overcome this issue, *LCB4* gene encoding major LCB kinase was deleted (*lcb2∆ lcb4∆*). These mutations enable achieving structural replacement of yeast LCBs with exogenously supplied SPH [9, 10] (Fig. 1B). The exogenously supplied SPH was metabolized into Cers and complex sphingolipids; however, cells with the SPH replacement exhibited abnormal membrane properties and hypersensitivity to multiple stresses [9, 10]. These findings suggest that, in *S. cerevisiae*, structural replacement of LCB to heterologous type causes abnormal phenotypes.

In this study, we explored the structural variations of LCBs that can be supplied to LCB biosynthesis‐deficient yeast. As a result, it was found that *S. cerevisiae* can grow even when its sphingolipid LCB moiety is replaced by t18:1(8*Z*) or d18:2(4*E*8*E*). To our knowledge, this is the first study to establish a eukaryotic cell in which sphingolipids are almost entirely composed of LCB with a *cis* double bond (t18:1(8*Z*)) as their backbone. The yeast cells with various LCB structure replacements are expected to serve as valuable tools for investigating the structure–function relationships of sphingolipids, which may contribute to understanding their evolutionary diversity.

## Materials and methods

### Materials

Phytosphingosine (t18:1) (PHS) was purchased from FOCUS Biomolecules (Plymouth Meeting, PA, USA). Sphingosine (d18:1) (SPH) was from Cayman Chemical (Ann Arbor, MI, USA). (4*E*,14*Z*)‐sphinga‐4,14‐dienine (d18:2(4*E*,14*Z*)) was from Avanti Polar Lipids (Alabaster, AL, USA). (8*Z*)‐4‐hydroxy‐8‐sphingenine (t18:1(8*Z*)), (4*E*,8*E*)‐sphinga‐4,8‐dienine (d18:2(4*E*8*E*)), (4*E*,8*Z*)‐sphinga‐4,8‐dienine (d18:2(4*E*8*Z*)), and 9‐methyl‐(4*E*,8*E*)‐sphinga‐4,8‐dienine (d19:2(4*E*,8*E*) (9Me)) were from Nagara Science (Gifu, Japan). All other reagents were of the highest purity available.

### Yeast strains and media

The *S. cerevisiae* strains used in this study are listed in Table 1. To tag the C terminus of Pil1, Pma1, or Pho88 with yeast‐enhanced green fluorescent protein (yeGFP), a yeGFP fusion cassette containing the *kanMX4* marker from pKT127 was inserted immediately upstream of the stop codon of each chromosomal gene, as described in a previous study [13]. To obtain *lcb2∆ lcb4∆ sur2∆* cells expressing yeGFP‐tagged Pil1, Pma1, and Pho88, random spore analysis was performed according to the method in [14], using YKY73 (BY4741 (*MATa*) *lcb2Δ::URA3 lcb4Δ::natNT2 sur2Δ::hphNT1*) and THY117 (Y7092 (*MATα*) *PIL1‐yeGFP::kanMX4*), THY92 (Y7092 (*MATα*) *PMA1‐yeGFP::kanMX4*), or THY129 (Y7092 (*MATα*) *PHO88‐yeGFP::kanMX4*). Briefly, these cells were mated, and the resulting diploids underwent sporulation before being subjected to random spore analysis. The haploid selection medium was SC/MSG (0.17% yeast nitrogen base without amino acids and ammonium sulfate (BD Difco, Heidelberg, Germany), 0.1% L‐glutamic acid sodium salt hydrate (MSG; Sigma‐Aldrich, St. Louis, MO, USA), 2% glucose, and nutritional supplements) lacking histidine, arginine, lysine, and uracil, but containing 25 μg·mL^−1^ canavanine, 25 μg·mL^−1^ thialysine, 200 μg·mL^−1^ G418, 100 μg·mL^−1^ clonNAT, 300 μg·mL^−1^ hygromycin B, 15 μm PHS, and 0.0015% Nonidet P40. When MTY174 (BY4741, *URA3*) and THY117, THY92, or THY129 were used, the haploid selection medium was SC/MSG lacking histidine, arginine, lysine, and uracil, but containing 25 μg·mL^−1^ canavanine, 25 μg·mL^−1^ thialysine, and 200 μg·mL^−1^ G418.

**Table 1:** Strains used in this study.

Strain	Genotype	Source
BY4741	*MATa his3∆1 leu2∆0 met15∆0 ura3∆0*	[34]
Y7092	*MATα can1∆::STE2pr‐his5 lyp1∆ his3∆1 leu2∆0 met15∆0 ura3∆0*	[14]
YKY73	BY4741, *lcb2Δ::URA3 lcb4Δ::natNT2 sur2Δ::hphNT1*	[10]
YKY81	BY4741, *lcb2Δ::LEU2 lcb4Δ::natMX4 sur2Δ::URA3 tetO*_*7*_ *‐LIP1::kanMX4*	[10]
MTY128	BY4741, *erg6Δ::kanMX4*	[19]
THY117	Y7092, *PIL1‐yeGFP::kanMX4*	This study
THY92	Y7092, *PMA1‐yeGFP::kanMX4*	This study
THY129	Y7092, *PHO88‐yeGFP::kanMX4*	This study
MTY174	BY4741, *URA3*	[18]
THY121	BY4741/Y7092, *MATa lcb2Δ::URA3 lcb4Δ::natNT2 sur2Δ::hphNT1 PIL1‐yeGFP::kanMX4*	This study
THY105	BY4741/Y7092, *MATa lcb2Δ::URA3 lcb4Δ::natNT2 sur2Δ::hphNT1 PMA1‐yeGFP::kanMX4*	This study
THY131	BY4741/Y7092, *MATa lcb2Δ::URA3 lcb4Δ::natNT2 sur2Δ::hphNT1 PHO88‐yeGFP::kanMX4*	This study
THY122	BY4741/Y7092, *MATa PIL1‐yeGFP::kanMX4 URA3*	This study
THY123	BY4741/Y7092, *MATa PMA1‐yeGFP::kanMX4 URA3*	This study
THY133	BY4741/Y7092, *MATa PHO88‐yeGFP::kanMX4 URA3*	This study

### Culture of LCB‐deficient cells with LCBs

*lcb2∆ lcb4∆ sur2∆* cells were cultured in YPD medium (1% yeast extract, 2% peptone and 2% glucose) containing 5 μm LCB, 0.0015% Nonidet P40 (dispersant), and 0.5% ethanol (vehicle). Alternatively, the cells were cultured on YPD agar plates containing 15 μm PHS, 0.0015% Nonidet P40, and 0.35% ethanol. For analysis of the time course of cell growth, cells were cultured overnight in YPD medium containing 5 μm PHS at 30 °C. Then, cells were resuspended in fresh YPD containing 5 μm each LCB at a concentration of 0.5 OD_600_ units per mL (unless otherwise specified). For analyses of lipids, fluorescence microscopy, flow cytometry, and zymolyase treatment, cells were first cultured in YPD medium containing 5 μm PHS for 8 h at 30 °C, diluted to 0.5 OD_600_ unit per mL in fresh YPD containing 5 μm each LCB, and then incubated overnight. After overnight incubation, cells were diluted to 0.5 OD_600_ unit per mL or 0.6 OD_600_ unit per mL (for lipid analysis) in fresh YPD containing 5 μm each LCB, and cultured for 8 h. The culture of wild‐type cells in all experiments was conducted without LCB.

### Lipid analysis by TLC and LC‐ESI MS/MS

Lipids were extracted as described previously [10, 15]. Briefly, for TLC analysis, cells (3 OD_600_ units (for detection of complex sphingolipids)) or 1.5 OD_600_ U (for detection of sterols) were suspended in 350 μL of ethanol/water/diethyl ether/pyridine/15 M ammonia (15 : 15 : 5 : 1 : 0.018, v/v) and then incubated at 65 °C for 15 min. The lipid extract was centrifuged at 10 000 ***g*** for 1 min and then extracted once more in the same manner. The resulting supernatants were dried. For analysis of complex sphingolipids but not sterols, the lipid extracts were dissolved in 130 μL monomethylamine (40% methanol solution)/water (10 : 3, v/v), incubated for 1 h at 53 °C (mild alkaline treatment), and then dried. The lipids were suspended in 60 μL of chloroform/methanol/water (5 : 4 : 1, v/v) and then separated on Silica Gel 60 TLC plates (Merck, Whitehouse Station, NJ) with chloroform/methanol/4.2 M ammonia (9 : 7 : 2, v/v) (for detection of complex sphingolipids) or hexane/diethyl ether/acetic acid (30 : 70 : 1, v/v) (for detection of sterols) as the solvent system. The TLC plates were sprayed with 10% copper sulfate in 8% orthophosphoric acid and then heated at 180 °C to visualize lipids. For LC‐ESI MS/MS analysis, 10 nmol of C6‐phytoceramide (Cayman) was added to cells (6 OD_600_ units) just before lipid extraction as an internal standard. Cers were measured by LC‐ESI MS/MS (3200 QTRAP; SCIEX, MA, USA) as described previously [10, 15].

### Incorporation of rhodamine 6G into cells

Evaluation of plasma membrane permeability by rhodamine 6G uptake into cells was performed as described previously with several modifications [16]. Briefly, cells (1 OD_600_ units) were resuspended in 300 μL of YPD medium containing 10 μm rhodamine 6G (Tokyo Chemical Industry, Tokyo, Japan) and then incubated for 30 min at 30 °C. The cells were collected by centrifugation, suspended with 700 μL of 10 mm NaN_3_ and NaF, and the fluorescence intensity of rhodamine 6G in each cell was measured by flow cytometry.

### di‐4‐ANEPPDHQ staining

Evaluation of plasma membrane lipid order by di‐4‐ANEPPDHQ was performed as described in [10] with several modifications. Briefly, cells (1 OD_600_ unit) were suspended in 100 μL of YPD medium containing 5 μm di‐4‐ANEPPDHQ (Potentiometric Probes, Farmington, CT, USA), incubated for 1 min at 30 °C, which enables staining of plasma membranes with the dye. Measurement of the intensity of green fluorescence (emission wavelength of 525 ± 20 nm) and red fluorescence (emission wavelength of 610 ± 10 nm), which was excited at 488 nm, in individual cells was performed by flow cytometry.

### Staining of cell‐wall components

Cells were harvested by centrifugation and fixed in 500 μL of 2% paraformaldehyde prepared in PBS (−) at room temperature for 30 min. Following two washes with PBS (−), the cells were incubated with either 10 μg·mL^−1^ calcofluor white (CFW; Sigma‐Aldrich) or 50 μg·mL^−1^ FITC‐conjugated concanavalin A (ConA‐FITC; Sigma‐Aldrich) for 60 min. After staining, the cells were then washed with PBS (−) and viewed under a fluorescence microscope.

### CMAC staining

Cells (1 OD_600_ units) were resuspended in 100 μL of PBS (−) containing 10 μm CellTracker™ Blue CMAC (Thermo Fisher Scientific, Waltham, MA, USA), and then incubated for 15 min at 30°C. Cells were washed twice with PBS(−) and viewed under a fluorescence microscope.

### Statistical analysis

Statistical analysis was performed using Student's *t*‐test, and the *P* values obtained are indicated. The Tukey–Kramer test was also used to assess multiple comparisons.

## Results

### Complementation of growth of LCB‐deficient yeast cells by addition of plant‐ or animal‐type LCBs

In this study, we investigated which animal‐ or plant‐derived LCBs can complement the growth of LCB‐deficient cells (*lcb2∆ lcb4∆ sur2∆* cells) by substituting for the yeast's endogenous LCBs (PHS and DHS). The deletion of *LCB2* was performed to block *de novo* synthesis of LCBs, and *LCB4* was deleted to prevent excessive accumulation of LCB 1‐phosphates derived from exogenously added LCBs, which can be cytotoxic [9, 10]. *SUR2*, which is involved in hydroxylation at the C‐4 position of LCBs, was also deleted to prevent the conversion of exogenously added DHS to PHS [9]; however, this mutation is not specifically required for the outcomes of this study. As shown in Fig. 1C, when various LCBs that are commercially available were added extracellularly to LCB‐deficient cells, the addition of PHS (t18:0), the major LCB in *S. cerevisiae*, resulted in the highest growth. As reported previously [9, 10], the growth complementation was also observed with the addition of SPH (d18:1), although it did not reach the level of growth observed with PHS. Interestingly, the addition of t18:1(8*Z*), a plant‐type LCB with a *cis* double bond in PHS, and d18:2(4*E*,8*E*), a plant‐type LCB with a *trans* double bond in SPH, also resulted in growth complementation; however, d18:2(4*E*,8*Z*) did not complement growth at all (Fig. 1B and C). The difference between d18:2(4*E*,8*E*) and d18:2(4*E*,8*Z*) suggests that the presence of a *trans* or *cis* double bond at the C8 position of SPH significantly influences its ability to complement growth in LCB‐deficient cells. Moreover, no growth complementation was observed with the addition of d18:2(4*E*,14*Z*), a sphingadiene from animals, or d19:2(4*E*,8*E*) (9Me), a methylated form of d18:2(4*E*,8*E*) (Fig. 1B and C). Since LCBs themselves can exhibit cytotoxicity in *S. cerevisiae* [17, 18], we examined the effects of each LCB on the growth of LCB‐deficient cells cultured with PHS (Fig. 1D). The addition of SPH, d18:2(4*E*,8E), d18:2(4*E*,8*Z*), or d19:2(4*E*,8*E*) (9Me) slightly inhibited the growth of LCB‐deficient cells cultured with PHS; however, d18:2(4*E*,14*Z*) strongly inhibited the cell growth (Fig. 1D). This suggests that the strong cytotoxicity of d18:2(4*E*,14*Z*) in *S. cerevisiae* may be one of the reasons why it fails to complement the growth of LCB‐deficient cells. In subsequent analyses, we focused on cells supplied with PHS, SPH, t18:1(8*Z*), or d18:2(4*E*,8*E*) (referred to as PHS, SPH, t18:1(8*Z*), or d18:2(4*E*,8*E*) cells).

**Fig. 2:**
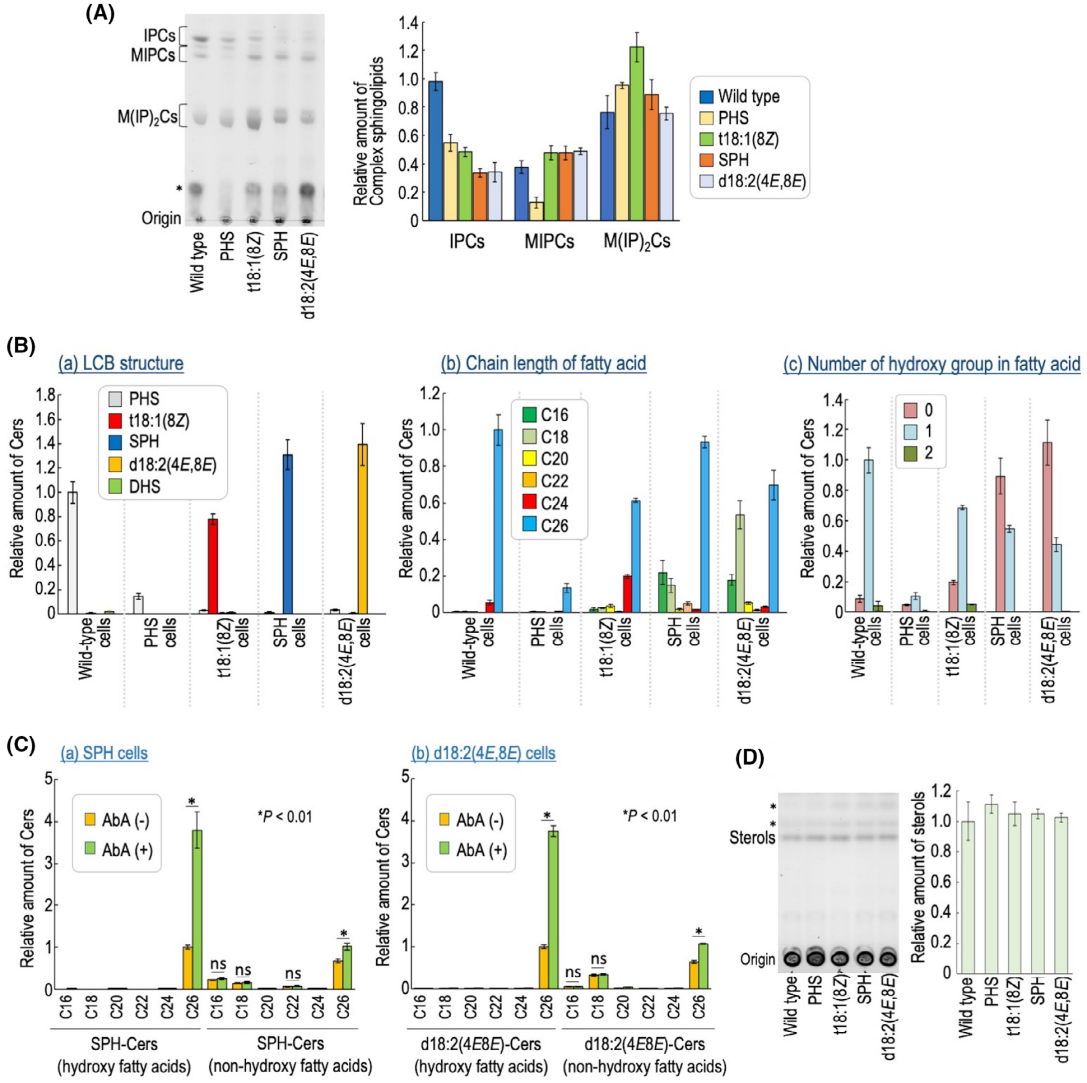
Lipid analysis of long‐chain base (LCB)‐replaced cells. (A) TLC analysis of complex sphingolipids. Lipids were extracted from wild‐type cells (without exogenously added LCB) or *lcb2∆ lcb4∆ sur2∆* cells cultured with each LCB. Lipids were extracted, subjected to mild alkaline treatment, separated by TLC, and then visualized with a copper sulfate and orthophosphoric acid reagent. The asterisk indicates unidentified bands. The amount of complex sphingolipids [IPCs, MIPCs, and M(IP)_2_Cs] in wild‐type cells was taken as 1. Data represent means ± SD for one experiment (triplicate) representative of three independent experiments. (B) Free Cer analysis by LC‐ESI MS/MS. As described in Fig. 2A, cells were cultured and lipids were extracted. Free Cer levels in each cell were measured using MRM mode, based on combinations of LCBs and fatty acids differing in chain length and hydroxylation state. The amounts of Cers are presented according to the LCB structure (panel a), fatty acid chain length (panel b), and fatty acid hydroxylation state (panel c). Data represent means ± SD for one experiment (triplicate) representative of three independent experiments. (C) Effect of inhibition of IPC synthesis by AbA on accumulation of free Cers. Before harvesting, cells were treated with 0 or 0.1 μg·mL^−1^ AbA for 1 h at 30 °C. Lipids were extracted, and free Cers were measured by LC‐ESI MS/MS. The amount of SPH‐Cer or d18:2(4*E*8*E*)‐Cer with hydroxylated C26‐fatty acid in untreated cells was taken as 1. Data represent means ± SD for one experiment (triplicate) representative of three independent experiments. Statistical analysis was performed using Student's *t*‐test, and the *P* values obtained are indicated. ns: no significant difference. (D) TLC analysis of sterols of wild‐type and LCB‐replaced cells. Data represent means ± SD for one experiment (triplicate) representative of three independent experiments. The details are given under Section 2.

Figure 2A shows the results of TLC analysis of complex sphingolipids, which were visualized with a copper sulfate and orthophosphoric acid reagent. In all LCB‐replaced cells, bands corresponding to inositol phosphorylceramides (IPCs), mannosylinositol phosphorylceramides (MIPCs), and mannosyldiinositol phosphorylceramides (M(IP)_2_Cs), which are subtypes of complex sphingolipids in yeast, were observed as in wild‐type cells; however, the quantitative balance of each subtype was somewhat different between cells (Fig. 2A). In addition, in SPH, t18:1(8*Z*), and d18:2(4*E*,8*E*) cells, Rf values of each subtype were slightly shifted compared with those in wild‐type and PHS cells, possibly due to differences in the LCB structure (Fig. 2A). Analysis of free Cer in each cell using LC‐ESI MS/MS revealed that more than 94% of the LCB structure within Cers was replaced by the extracellularly added LCB (Fig. 2B). The carbon chain length of fatty acids in sphingolipids is primarily C26 in budding yeast, and most of them are α‐hydroxylated fatty acids [2]. In all LCB‐replaced cells, Cers containing C26 fatty acids were most abundant, and the hydroxylated fatty acids were detected (Fig. 2B). However, in SPH and d18:2(4*E*,8*E*) cells, more than 18% of free Cers contained C16 or C18 fatty acids (Fig. 2B,C). In addition, in contrast to wild‐type, PHS, and t18:1(8*Z*) cells, in which Cers containing hydroxylated fatty acids were predominant, SPH and d18:2(4*E*,8*E*) cells showed higher levels of free Cers with nonhydroxylated fatty acids than those with hydroxylated ones (Fig. 2B). To examine whether the Cers containing either C16 or C18 fatty acids, or nonhydroxylated fatty acids, detected in SPH and d18:2(4*E*,8*E*) cells were converted into complex sphingolipids, SPH and d18:2(4*E*,8*E*) cells were treated with aureobasidin A (AbA), which inhibits the conversion of Cers to IPCs (Figs 1A and 2C). In SPH cells, AbA treatment caused an increased level of free Cers containing SPH and hydroxylated C26 fatty acid (Fig. 2C). Similarly, an increase was also observed in free Cers containing SPH and nonhydroxylated C26 fatty acid, although the extent of the increase was smaller than that of Cers with hydroxylated C26 fatty acid. However, such an increase was not observed in Cers containing SPH and C16 or C18 fatty acid. These trends are consistent with a previous report [10]. In d18:2(4*E*,8*E*) cells as well, Cers containing d18:2(4*E*,8*E*) and C16 or C18 fatty acids did not increase upon AbA treatment, whereas those with hydroxylated C26 fatty acids showed the most pronounced increase (Fig. 2C). These results suggest that, in SPH and d18:2(4*E*,8*E*) cells, Cers containing shorter chain fatty acids or nonhydroxylated fatty acids are less likely to be utilized for the biosynthesis of complex sphingolipids. We also examined the cellular level of sterols, which are functionally related to sphingolipids [15, 19, 20]; however, notable changes were not observed in any of the LCB‐replaced cells (Fig. 2D).

**Fig. 3:**
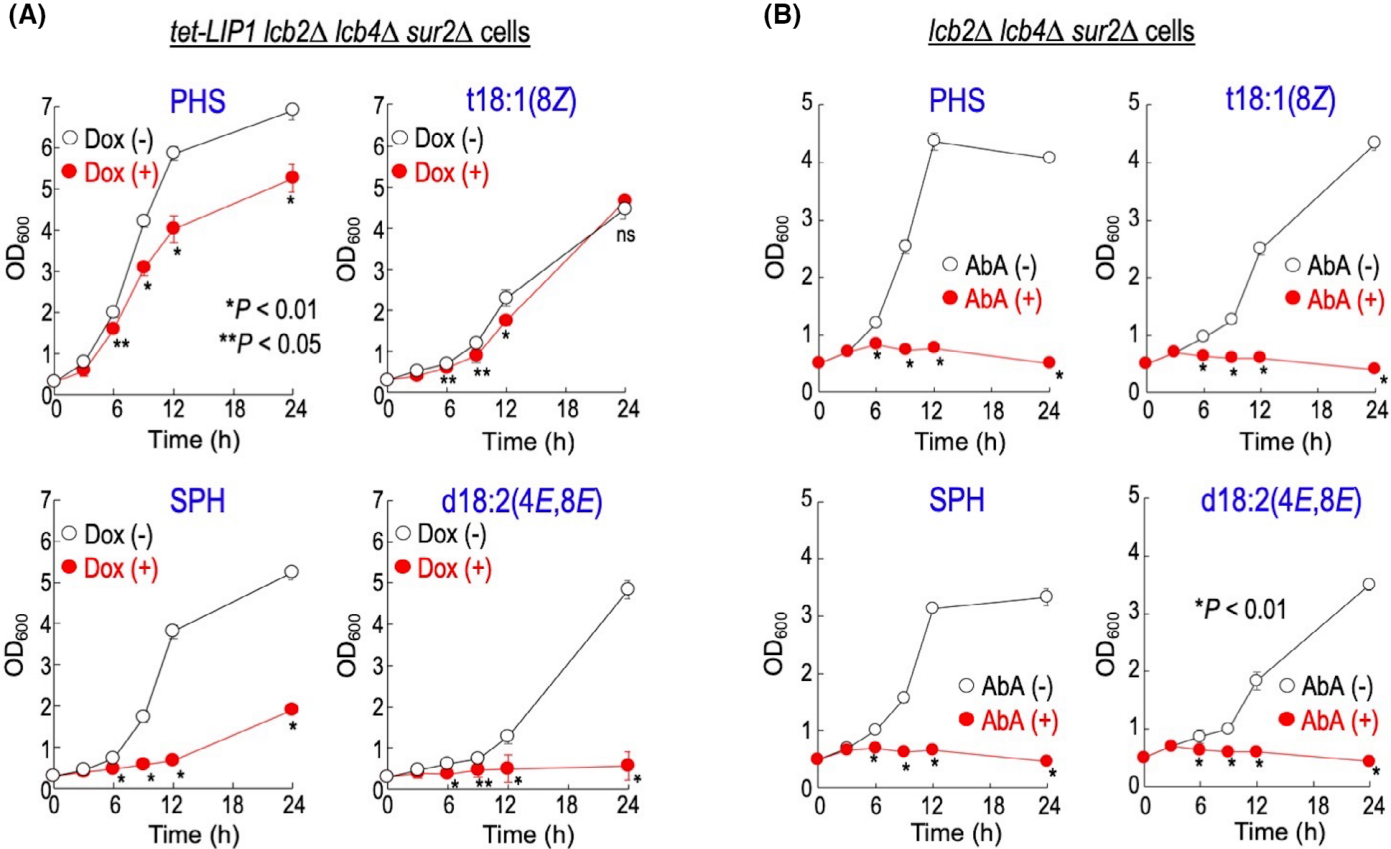
Effects of repression of Cer and complex sphingolipid biosynthesis on growth of long‐chain base (LCB)‐replaced cells. (A) Effect of Cer synthesis in LCB‐replaced cells. *tet‐LIP1 lcb2∆ lcb4∆ sur2∆* cells were cultured overnight in YPD medium containing 5 μm phytosphingosine (PHS) at 30 °C. Then, cells (0.3 OD_600_ units per mL) were resuspended in fresh YPD containing 5 μm each LCB and 0 or 10 μg·mL^−1^ doxycycline (Dox), incubated at 30 °C, and aliquots of cell suspensions were subjected to cell density measurements (OD_600_) at the indicated times. (B) Effect of Aureobasidin A (AbA) in LCB‐replaced cells. *lcb2∆ lcb4∆ sur2∆* cells were cultured overnight in YPD medium containing 5 μm PHS at 30 °C. Then, cells (0.5 OD_600_ units per mL) were resuspended in fresh YPD containing 5 μm each LCB and 0 or 0.1 μg·mL^−1^ AbA, incubated at 30 °C, and aliquots of cell suspensions were subjected to OD_600_ measurements at the indicated times. Data represent means ± SD for one experiment (triplicate) representative of three independent experiments. Statistical analysis was performed using Student's *t*‐test, and the *P* values obtained are indicated. ns: no significant difference. The details are given under Section 2.

To investigate whether the metabolism of the extracellularly added LCBs into Cer or beyond is necessary for the growth of LCB‐replaced cells, we utilized an LCB‐deficient cell in which the Cer synthase regulatory subunit gene *LIP1* (Fig. 1A) is expressed under a tetracycline‐regulated promoter, allowing repression of *LIP1* expression by doxycycline (Dox) (*tet‐LIP1 lcb2∆ lcb4∆ sur2∆* cells) [10, 21]. As shown in Fig. 3A, repression of *LIP1* by Dox treatment caused a growth delay in all LCB‐replaced cells, although the extent of delay varied. In addition, treatment with AbA led to a strong growth defect in all LCB‐replaced cells (Fig. 3B). These results suggested that, in the LCB‐replaced cells, further metabolic conversion of the supplemented extracellular LCBs is necessary for maintenance of growth. AbA not only decreases levels of complex sphingolipids but also leads to the accumulation of Cers, both of which contribute to the growth inhibition associated with this compound [21]. Thus, it should also be considered the possibility that causes of the growth inhibition observed in the LCB‐replaced cells with AbA treatment also include accumulation of Cers.

### Effect of structural replacement of LCBs on properties of plasma membranes and cell walls

**Fig. 4:**
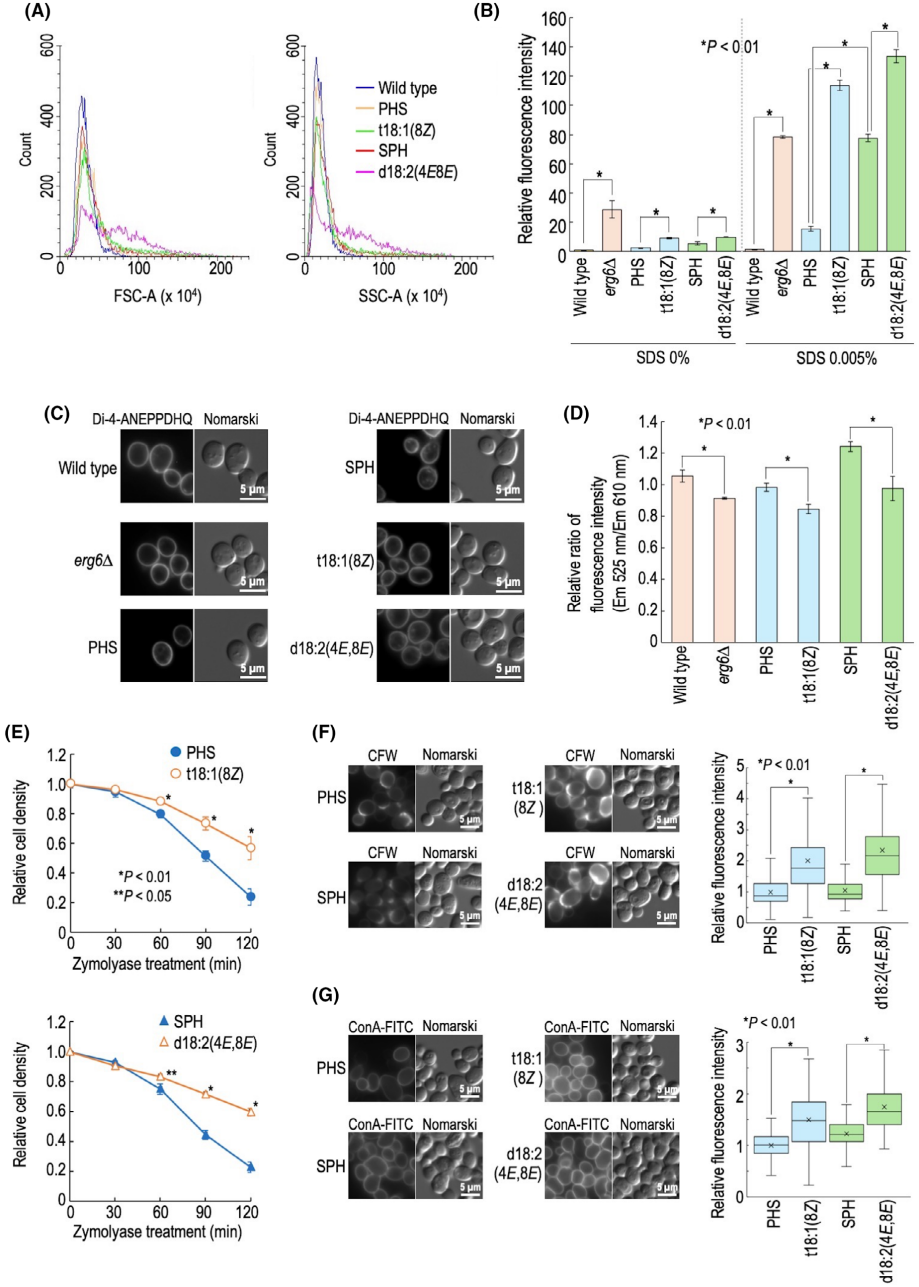
Properties of plasma membranes and cell walls in long‐chain base (LCB)‐replaced cells. (A) The forward scatter (FSC‐A) and side scatter (SSC‐A) for *lcb2∆ lcb4∆ sur2∆* cells cultured with each LCB were measured by flow cytometry. (B) Incorporation efficiency of rhodamine 6G into cells in the presence or absence of SDS. Wild‐type and *erg6∆* cells, and *lcb2∆ lcb4∆ sur2∆* cells cultured with each LCB (1 OD_600_ unit) were suspended in 300 μL of YPD containing 0 or 0.005% SDS, and then incubated with 10 μm rhodamine 6G for 30 min at 30 °C, and the incorporated rhodamine 6G into cells was measured by flow cytometry. Each value represents the average fluorescence intensity of 10 000 cells from a single sample. The fluorescence intensity in wild‐type cells without SDS was taken as 1. Data represent means ± SD for three samples representative of three independent experiments. Statistical analysis was performed using the Tukey–Kramer test, and the *P* values obtained are indicated. (C) Plasma membrane staining by di‐4‐ANEPPDHQ. Wild‐type and *erg6∆* cells, and *lcb2∆ lcb4∆ sur2∆* cells cultured with each LCB (1 OD_600_ unit) were suspended in 100 μL of YPD medium containing 5 μm di‐4‐ANEPPDHQ, and then incubated for 1 min at 30 °C. Cells were then washed with water, and green fluorescence was observed under a fluorescence microscope. (D) Evaluation of membrane lipid order by using di‐4‐ANEPPDHQ. Cells were stained with di‐4‐ANEPPDHQ for 1 min at 30 °C. The ratio of green (525 nm) and red (610 nm) fluorescence in individual cells was measured with a flow cytometer. Each value represents the average fluorescence ratio of 10 000 cells from a single sample. The fluorescence intensity in wild‐type cells was taken as 1. Data represent means ± SD for three samples representative of three independent experiments. Statistical analysis was performed using Student's *t*‐test, and the *P* values obtained are indicated. (E) Zymolyase sensitivity. *lcb2∆ lcb4∆ sur2∆* cells cultured with each LCB were washed with 20 mm HEPES buffer (pH 7.5), resuspended (1.5 OD_600_ units per mL) in the same buffer containing 15 μg·mL^−1^ zymolyase‐20 T (Nacalai Tesque), and then incubated at 30 °C. At the indicated times, the cell density (OD_600_) in the cell suspensions was measured. Data represent means ± SD for three samples representative of three independent experiments. Statistical analysis was performed using Student's *t*‐test, and the *P* values obtained are indicated. (F and G) Cell wall component staining. Cells were fixed, stained with CFW (F) or concanavalin A (ConA)‐FITC (G), and then viewed under a fluorescence microscope. The fluorescence intensity of individual cells is expressed as boxplots with median, interquartile range (25th–75th percentile), and whiskers representing full range excluding outliers. Data represent the value for 100 cells for individual strains. The average (marked as x) fluorescence intensity in wild‐type cells was taken as 1. Statistical analysis was performed using Student's *t*‐test, and the *P* values obtained are indicated. The details are given under Section 2.

To obtain morphological information on the LCB‐replaced cells, forward scatter (FSC) and side scatter (SCC) profiles were examined using flow cytometry (Fig. 4A). Notable differences in the profiles were not observed between PHS, SPH, and t18:1(8Z) cells; however, d18:2(4*E*,8*E*) cells exhibited different profiles, which may reflect some morphological abnormalities (Fig. 4A). Previous reports suggested that abnormalities in sphingolipids affect the integrity of plasma membranes and cell walls [15, 19]. Thus, we first examined the permeability of plasma membranes by measuring the incorporation of a lipophilic fluorescent dye rhodamine 6G into cells [15, 22]. As shown in Fig. 4B, in *ERG6*‐deleted cells, which were used as a positive control exhibiting increased plasma membrane permeability [10, 15], clear intracellular accumulation of the dye was observed. *ERG6* is involved in ergosterol biosynthesis, and *erg6∆* causes impaired integrity of plasma membranes [15]. Compared with PHS cells, cells where LCBs were replaced with t18:1(8*Z*), a modified form of PHS with a *cis* double bond, showed an increase in plasma membrane permeability. A similar trend was observed in cells replaced with d18:2(4*E*,8*E*), a modified form of SPH with a *trans* double bond, when compared with SPH cells (Fig. 4B). SDS directly damages plasma membranes, and we have previously reported that the accumulation of rhodamine 6G in SPH cells dramatically increases in the presence of 0.005% SDS compared with PHS cells [10] (Fig. 4B). In d18:2(4*E*,8*E*) cells, in the presence of SDS, rhodamine 6G accumulation was further enhanced compared with SPH cells. The accumulation in t18:1(8*Z*) cells was also much higher than in PHS cells in the presence of SDS (Fig. 4B). Thus, these results suggested that the replacement of LCBs with those containing double bonds in the C8 position exacerbates the detrimental effects of SDS on plasma membrane permeability. To further investigate the physical characteristics of plasma membranes, we utilized di‐4‐ANEPPDHQ, a probe for evaluating membrane lipid order [10, 15, 23]. Upon staining wild‐type, *erg6∆*, PHS, SPH, t18:1(8*Z*), d18:2(4*E*,8*E*) cells with di‐4‐ANEPPDHQ for 1 min, fluorescence was predominantly observed at the cell surface (Fig. 4C). di‐4‐ANEPPDHQ exhibits green fluorescence when residing in the ordered phase of membranes and red fluorescence in the disordered phase [10, 15, 23]. Flow cytometric analysis was conducted to quantify the ratio of green (525 nm) to red (610 nm) fluorescence (Fig. 4D). As reported previously [10, 15, 23], *erg6∆* cells displayed a reduced green‐to‐red fluorescence ratio compared with wild‐type cells, indicating a decrease in lipid order (Fig. 4D). Compared with PHS cells, t18:1(8*Z*) cells showed a decrease in lipid order. A similar trend was observed in SPH and d18:2(4*E*,8*E*) cells (Fig. 4D). These results support the notion that the addition of double bonds in the C8 position of LCBs leads to an increase in plasma membrane abnormalities (Fig. 4D). In contrast, an evaluation of cell wall integrity based on sensitivity to the cell wall‐digesting enzyme zymolyase revealed that, compared with PHS cells, t18:1(8*Z*) cells exhibited resistance to zymolyase (Fig. 4E). A similar trend was also observed in SPH cells and d18:2(4*E*,8*E*) cells (Fig. 4E). These results suggest that cell walls become more rigid when cellular LCB is replaced with LCBs containing a double bond at the C8 position. In Fig. 3F and G, PHS, SPH, t18:1(8Z), and d18:2(4*E*,8*E*) cells were stained with calcofluor white or FITC‐labeled concanavalin A (ConA), which bind to chitin and to alpha‐mannose in cell‐wall proteins containing mannan‐type *N*‐glycans, respectively. The fluorescence intensities of both CFW and ConA‐FITC were higher in t18:1(8*Z*) cells than in PHS cells (Fig. 4F and G), possibly reflecting increased amounts of chitin and cell‐wall mannans at the cell surface. A comparable increase in fluorescence intensity was also observed in SPH cells and d18:2(4*E*,8*E*) cells (Fig. 4F and G). These results likely reflect an increase in cell wall rigidity, as indicated by the zymolyase resistance (Fig. 4E). The exact reason for the increased cell wall rigidity in t18:1(8*Z*) and d18:2(4*E*,8*E*) cells remains unclear, but it is presumed that cells compensate for the impaired integrity of plasma membranes caused by LCB structural replacement through enhanced reinforcement of cell walls. Previously, we showed that mutant yeast cells lacking complex sphingolipid structural diversity (*csg1Δ csh1Δ sur2Δ scs7Δ*) also exhibit compromised plasma membrane integrity [15]. However, when the general stress‐responsive transcription factor genes *MSN2* and *MSN4* are deleted, cells lacking complex sphingolipid structural diversity display increased resistance to zymolyase [15]. These findings support the notion that defects in plasma membrane integrity caused by sphingolipid abnormalities are compensated for by the reinforcement of cell walls.

### Abnormal distribution pattern of Pil1 and Pma1 in LCB‐replaced cells

**Fig. 5:**
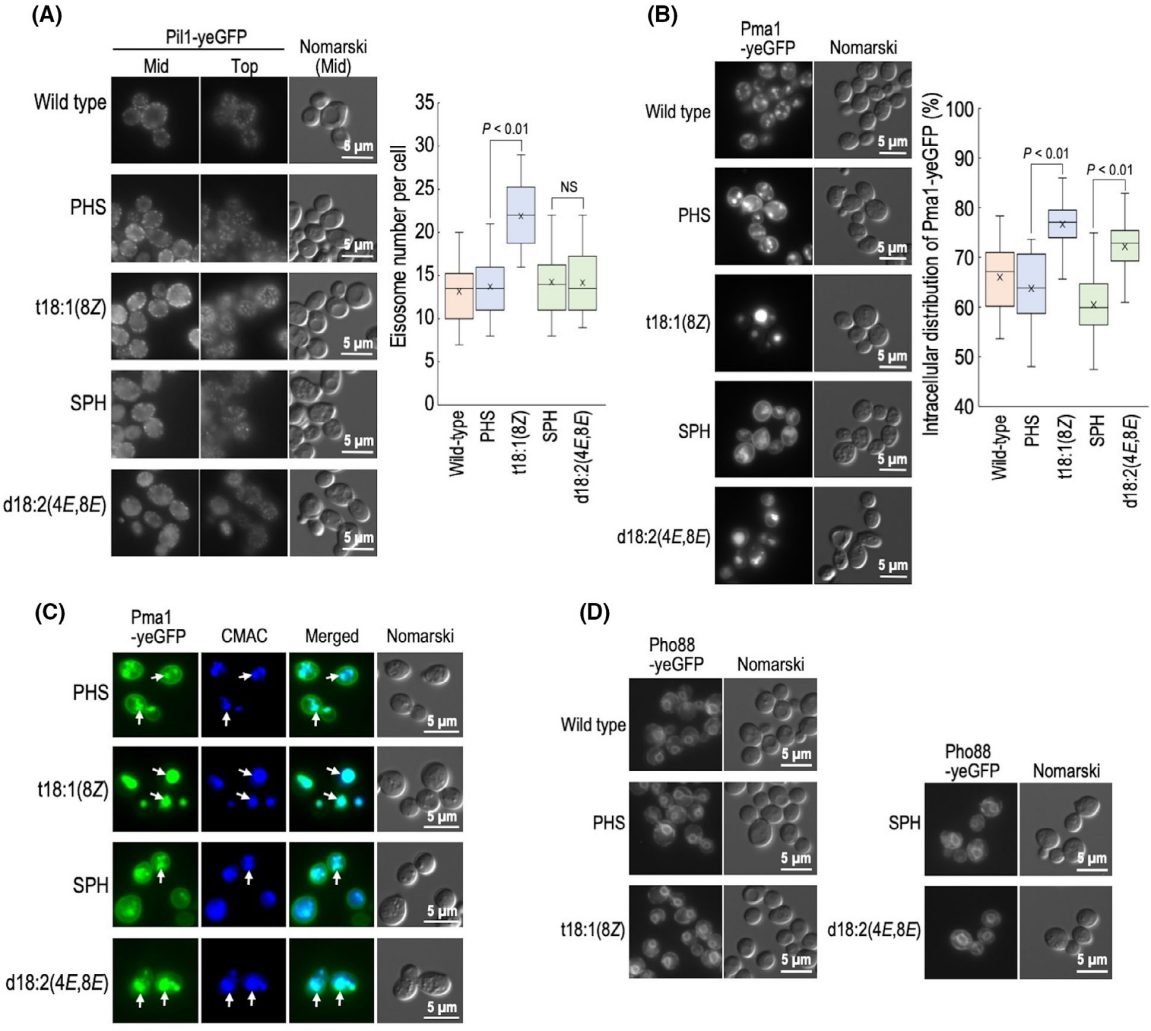
Localization of membrane‐associated proteins in long‐chain base (LCB)‐replaced cells. (A) Distribution of Pil1‐yeGFP in LCB‐replaced cells. Wild‐type cells expressing Pil1‐yeGFP and *lcb2∆ lcb4∆ sur2∆* cells, which were cultured with each LCB and expressing Pil1‐yeGFP, were observed under a fluorescence microscope. Eisosomes (dot structure) in the top sections of images of individual cells (*n* = 30) were counted using imagej software (NIH), and the number of eisosomes was expressed as boxplots with median, interquartile range (25th–75th percentile), and whiskers representing full range excluding outliers. Statistical analysis was performed using Student's *t*‐test, and the *P* values obtained are indicated. (B) Distribution of Pma1‐yeGFP in LCB‐replaced cells. Wild‐type cells expressing Pma1‐yeGFP and *lcb2∆ lcb4∆ sur2∆* cells, which were cultured with each LCB and expressing Pma1‐yeGFP, were observed under a fluorescence microscope. The intracellular distribution of Pma1‐yeGFP is defined as the total cellular fluorescence minus the plasma membrane fluorescence, divided by the total cellular fluorescence, expressed as a percentage (*n* = 30). Data are presented as boxplots with median, interquartile range (25th–75th percentile), and whiskers representing full range excluding outliers. Statistical analysis was performed using Student's *t*‐test, and the *P* values obtained are indicated. (C) *lcb2∆ lcb4∆ sur2∆* cells cultured with each LCB expressing Pma1‐yeGFP were stained with CellTracker™ Blue CMAC, and then observed by fluorescent microscopy. (D) Wild‐type cells expressing Pho88‐yeGFP and *lcb2∆ lcb4∆ sur2∆* cells, which were cultured with each LCB and expressing Pho88‐yeGFP, were observed under a fluorescence microscope. The details are given under Section 2.

Sphingolipids are important for the formation of plasma membrane microdomains [1]. Thus, we next observed the GFP fusion proteins of Pil1 and Pma1, which localize to distinct typical yeast microdomains [24, 25, 26]. Pil1 is a core protein of eisosomes, which include MCC (Membrane Compartment of Can1) [24, 25]. Eisosomes are microdomains with a furrow‐like morphology on plasma membranes and are detected as dot‐like structures when observed with Pil1‐yeGFP [25]. As shown in Fig. 5A, there was no significant difference in the number of Pil1‐yeGFP dots on plasma membranes in wild‐type, PHS, SPH, and d18:2(4*E*,8*E*) cells; however, an increase in the number of dots was observed in t18:1(8*Z*) cells, suggesting some abnormalities in eisosomes. Since previous studies have shown that hyperosmotic stress, which causes cell shrinkage and alters plasma membrane tension, can affect eisosome abundance [27, 28], the increased number of dots in t18:1(8*Z*) cells may also indicate not only abnormalities in eisosome organization but also broader alterations in plasma membrane properties. MCP (Membrane Compartment of Pma1) is another microdomain in *S. cerevisiae* [26]. In wild‐type, PHS, and SPH cells, Pma1‐yeGFP was observed on plasma membranes and inside the cells; however, in t18:1(8*Z*) and d18:2(4*E*,8*E*) cells, the plasma membrane distribution was decreased, and instead, abnormal accumulation inside the cells was observed (Fig. 5B). The intracellular accumulation of Pma1 was confirmed to occur in vacuoles, as indicated by vacuole staining with Cell Tracker Blue (CMAC) (Fig. 5C). In contrast, in any of the LCB‐replaced cells, notable abnormalities were not observed in Pho88‐yeGFP, an endoplasmic reticulum‐localized membrane protein [29] (Fig. 5D), suggesting that not all membrane‐bound proteins exhibit abnormal distribution in LCB‐replaced cells.

## Discussion

Generally, the majority of sphingolipids in eukaryotic cells lack *cis* double bonds in their LCBs [3], and in *S. cerevisiae*, sphingolipids with *trans* double bonds are also absent [2]. The presence of *cis* double bonds in the hydrophobic acyl chains of membrane lipids introduces kinks, disrupting tight packing and thereby enhancing membrane fluidity [30]. In contrast, sphingolipids typically contain saturated or mostly straight‐chain hydrocarbons in their hydrophobic regions, which contribute to the ordered structure of biological membranes. Notably, in mammalian cells, sphingolipids with d18:2(4*E*,14*Z*) are suggested to be excluded from microdomain formation [6]. To our knowledge, there have been no reports of eukaryotic cells in which nearly all sphingolipids in the cell possess LCBs containing *cis* double bonds. For example, in *Arabidopsis thaliana*, it has been shown that sphingolipids containing t18:1(8*Z*) account for less than 30% of the total sphingolipid content [4]. In this study, we found for the first time that yeast cells whose LCBs are almost entirely replaced with *cis* double bond‐containing LCB t18:1(8*Z*) can grow despite exhibiting abnormalities in plasma membrane properties. This experimental system provides a unique and valuable model for investigating the structure–function relationship of LCBs in membrane biology.

The abnormalities in proteins localized to lipid microdomains, including MCC and MCP, in the LCB‐replaced cells may be related to ergosterol, which physically interacts with sphingolipids in microdomain formation [1]. Previously, we reported that, when both LCB and sterols were simultaneously replaced with SPH and cholesterol, the cells with SPH and cholesterol exhibited more severe plasma membrane abnormalities and higher stress sensitivities than those with SPH and ergosterol or PHS and cholesterol [10]. Both t18:1(8*Z*) and d18:2(4*E*,8*E*) are plant‐derived LCBs, and plants contain beta‐sitosterol, campesterol, stigmasterol, and brassicasterol, all of which do not exist in *S. cerevisiae* [31]. The structural compatibility of these sterols with sphingolipids containing each LCB structure *in vivo* remains unknown. Future studies involving simultaneous replacement of t18:1(8*Z*), d18:2(4*E*,8*E*), and these sterols in budding yeast may provide new insights into the cooperative functions of sphingolipid‐sterol interactions, which are based on the microstructure of these lipids.

The LCB replacement system has potential applications in biotechnology and pathology. For example, yeast strains with endogenous LCBs replaced by specific structures could biosynthesize Cers and complex sphingolipids with desired features, useful in cosmetics, functional foods, and pharmaceuticals. Furthermore, applying this system to pathogenic fungi like *Candida albicans* could shed light on the role of sphingolipid structure in virulence, drug resistance, and membrane function. For example, given that *C. albicans* forms highly drug‐resistant biofilms and that sphingolipid biosynthesis influences biofilm formation [32, 33], the present system provides a useful tool for investigating how specific LCB structures affect biofilm development and membrane‐associated pathogenic traits.

## Conflicts of interest

The authors declare no conflict of interest.

## Author contributions

MT, TH, and SS conceived and coordinated the study, and wrote the paper. MT, TH, and SS designed and performed the analysis presented in Figs 1, 2, 3, 4, 5. YI performed the LC–MS/MS analysis. YK and HY were involved in the generation of the mutant cells used in this study. All authors read and approved the final version of the manuscript.

## Data Availability

The data that support the findings of this study are available from the corresponding author upon reasonable request.
